# Persian Paradigm for Language Cortical Mapping: Development, Feasibility, and Evaluation

**DOI:** 10.1002/brb3.71324

**Published:** 2026-03-31

**Authors:** Alireza Tabibkhooei, Maryam Jalali, Vahid Valinejad, Ehsan Shekari, Mahdi Mohammadi, Zeinab Oghabian, Pooya Derakhshan, Mohammad Javad Abdolhay, Mohammad Ali Oghabian

**Affiliations:** ^1^ Skull Base Research Center, Department of Neurosurgery Iran University of Medical Sciences Tehran Iran; ^2^ Department of Medical Physics and Biomedical Engineering School of Medicine Tehran University of Medical Sciences Tehran Iran; ^3^ Neuroimaging and Analysis Group, Research Center for Molecular and Cellular Imaging, Advanced Medical Technologies and Equipment Institute Tehran University of Medical Sciences Tehran Iran; ^4^ Pediatric Neurorehabilitation Research Center University of Social Welfare and Rehabilitation Sciences Tehran Iran; ^5^ Department of Speech Therapy, School of Rehabilitation University of Social Welfare and Rehabilitation Sciences Tehran Iran; ^6^ Department of Neuroscience, Advanced Technologies Faculty Iran University of Medical Sciences Tehran Iran; ^7^ Department of Molecular Imaging Faculty of Advanced Technologies in Medicine Iran University of Medical Sciences Tehran Iran; ^8^ Advanced Diagnostic and Interventional Radiology Research Center (ADIR) Tehran University of Medical Science Tehran Iran; ^9^ Faculty of Electrical Engineering Shahid Beheshti University Tehran Iran; ^10^ Department of Anesthesiology and Pain Medicine, School of Medicine Iran University of Medical Sciences Tehran Iran; ^11^ Rasool Akram Hospital Iran University of Medical Sciences Tehran Iran

**Keywords:** awake craniotomy, brain mass lesion, direct cortical stimulation, functional magnetic resonance imaging, language paradigm

## Abstract

**Background:**

Intraoperative language tasks often inadequately assess complex grammatical processes. This study aims to improve Persian cortical mapping by integrating object naming and sentence completion tasks. Multi‐task language (MTL), consisted of an object naming task and a semantically relevant verb, was designed.

**Methods:**

The content validity ratio (CVR) and index (CVI) were calculated to determine task items retention. Twenty‐five right‐handed patients with brain mass lesions in language‐associated regions were enrolled. Each patient received a medical recommendation for awake surgery, and preoperative fMRI data were collected through verb and syntax generation tasks. Activations in language‐related regions for object naming, word generation, and MTL were compared. Lateralization of brain activity was evaluated for each participant by calculating the Laterality Index (LI).

**Results:**

The CVR for all items ranged from 0.9 to 1 (0.93 ± 0.09), while the CVI ranged from 0.8 to 1 (0.94 ± 0.07). Significant activations were identified during object naming, particularly in the left precentral gyrus and inferior frontal gyrus (*z* = 7.53). For word generation, activations were bilateral in the fusiform gyrus and Broca (*z* = 5.54). The MTL task showed its highest activation in the fusiform and inferior temporal gyrus (*z* = 6.86). A significant difference was found between the LI of object naming and word generation (*p* = 0.003), while MTL comparisons were not statistically significant.

**Conclusion:**

The developed Persian intraoperative language paradigm, named MTL, demonstrated validity and feasibility, with significant activation in key language areas, particularly the left inferior frontal gyrus.

## Graphical abstract

Multi‐task language paradigm for Persian intraoperative mapping.

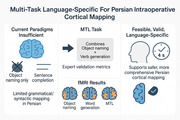

## 1 Introduction

Awake craniotomy with language cortical mapping is the gold standard for safely resecting brain masses in language‐critical areas, minimizing postoperative functional deficits (Bu et al. 2021; Hervey‐Jumper et al. 2015; Mcgirt et al. 2009). Surgery for lesions near motor or language regions requires accurate identification of involved cortical and subcortical structures (Jiménez de la Peña et al. 2013).

Various intraoperative language paradigms, such as object naming (Ojemann et al. 2002; Sanai and Berger 2009), sentence completion (Unadkat et al. 2019), and action naming (Bello et al. 2006; Bello et al. 2007; Bello et al. 2008), focus on specific aspects of language processing, potentially inadequately reflecting a patient's overall language capabilities. Additionally, these tasks can be time‐consuming and exhibit considerable variability. The intraoperative object naming task, the most common, is sensitive to phonological retrieval but involves less linguistic processing than tasks like verb production or sentence completion, limiting its ability to assess complex grammatical, syntactic, lexical‐semantic, or morpho‐phonological processes (Rofes and Miceli 2014).

Using Functional magnetic resonance imaging (fMRI) for preoperative lateralization and localization of language‐critical areas with these tasks is standard practice for enhancing presurgical planning, optimizing extent of craniotomy, and understanding eloquent area architecture around lesions. fMRI studies further indicate that the object naming task may not effectively identify Broca's area in healthy subjects (Rau et al. 2007).

Incorporating a grammatical component into the object naming task may enhance sensitivity, especially in patients with frontal brain tumors, potentially identifying areas missed by lexical‐semantic tasks. The lesion's location may also influence the choice of intraoperative linguistic tasks. However, linguistic functions, including phonological, morphological, semantic, and syntactic processing, are generally governed by distributed neural networks rather than being restricted to a single brain region (Coello et al. 2013).

The intraoperative language task must be valid and structured to meet the requirements of direct cortical stimulation (DCS), with each language test limited to 4 s to minimize the risk of DCS‐induced seizures (Kayama 2012; Mandonnet et al. 2007). The design of operational language tasks is influenced by the need for complex tasks, which require more than 4 s to complete, especially to cover the various linguistic subtasks necessary for producing accurate language utterances. This contrasts with simpler paradigms like object naming or verb processing (Rosengarth et al. 2021).

This study aims to develop and evaluate a language paradigm for intraoperative cortical mapping in Persian by combining object naming and sentence completion tasks to assess language performance within a 4‐s timeframe, thereby aligning the task with the recommended temporal constraints for direct cortical stimulation and potentially reducing the risk of stimulation‐related complications (including seizures). Additionally, we will implement this framework alongside traditional tasks in patients undergoing preoperative fMRI to assess its advantages and applicability as a standard language task for Persian cortical mapping.

## 2 Materials and Methods

### 2.1 Ethical Statement

This study was approved by the Ethics Committee of the Iran University of Medical Sciences (IR.IUMS.FMD.REC.1402.098). Written informed consent was obtained from all participants prior to enrollment and data acquisition. All procedures were conducted in accordance with institutional ethical standards and the Declaration of Helsinki.

### 2.2 Language Paradigm

**FIGURE 1:**
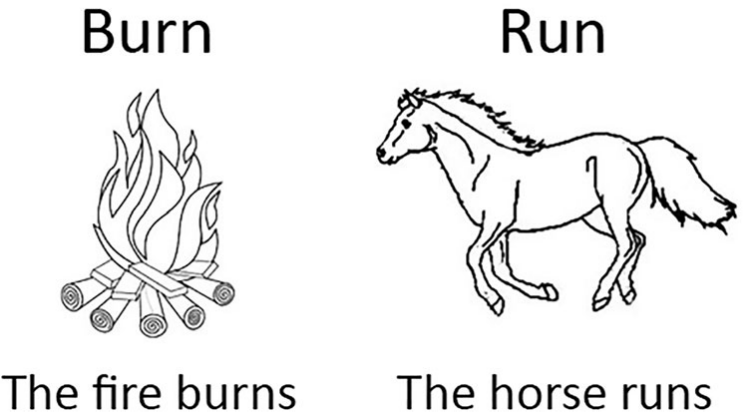
Two examples of picture–verb combinations used in the paradigm.

The Persian language paradigm for fMRI and intraoperative stimulation, named the multi‐task language (MTL), consists of an object naming task and a semantically relevant verb. Fifty black‐and‐white images were created, displaying objects centrally with verbs placed randomly above or below (Figure 1). Object names were drawn from the Persian naming test, while verbs were selected from frequently used words (Bijankhan and Mohseni 2012; Ghasisin et al. 2015). A linguist ensured matching in frequency, thematic structures, syllable count, and phonological complexity. Content validity was evaluated by 10 speech and language pathologists, who rated sentences as “essential,” “useful but not essential,” or “not necessary.” The content validity ratio (CVR) and index (CVI) were calculated to determine item retention, with items having a CVR > 0.62 and an I‐CVI > 0.79 being selected (Ahmad and Idris 2025).

The feasibility of the language task was tested to determine whether participants could finish it in under 4 s, reducing the risk of seizures during surgery. Printed stimuli were presented to 30 healthy individuals in random order, who were asked to create a grammatically and semantically correct sentence based on the image and verb. The tests were considered feasible when more than two‐thirds of participants successfully produced an appropriate sentence in under 4 s.

The finalized Persian stimuli used in the MTL paradigm are provided in Supplementary Table (original Persian language list) to facilitate use in Persian‐speaking clinical and research settings.

### 2.3 Patients

Twenty‐five right‐handed patients diagnosed with brain mass lesions located in language‐associated regions of the brain were enrolled in this study conducted from 2022 to 2024. Each patient received an independent medical recommendation for awake surgery. Preoperative fMRI data, collected through a series of verb and syntax generation tasks performed by the patients, indicated a close spatial relationship between critical language areas and the mass lesions. Language lateralization was assessed for all participants.

### 2.4 fMRI Data Acquisition and Analysis

All 25 participants were instructed to engage in a memory task while their blood oxygen level‐dependent (BOLD) responses, indicative of stimulus‐related changes, were recorded. Specifically, object naming tasks were administered to 17 patients, while 8 patients performed word generation tasks. A schematic representation of the task block designs is illustrated in Figure 1. Data acquisition was conducted using a 3T MRI scanner (Discovery MR750w; GE Healthcare; Milwaukee, USA). The parameters for the fMRI data, specifically for T2*‐weighted images, included 42 slices, a repetition time (TR) of 3000 ms, an echo time (TE) of 30 ms, a voxel size of 3 × 3 × 3 mm^3^, a flip angle of 90°, and a field of view of 240 × 240 mm^2^. To facilitate three‐dimensional visualization of the results, an additional T1‐weighted 3D inversion recovery‐prepared fast spoiled gradient‐recalled (SPGR) image was acquired, with parameters including TR = 8.176 ms, TE = 3.064 ms, a flip angle of 12°, and a slice thickness of 1 mm.

Image processing for language‐based fMRI was conducted using FSL software (Functional Magnetic Resonance Imaging of the Brain Software Library, Oxford, UK, version 6.0.0). The primary preprocessing steps included brain extraction, motion correction, spatial smoothing with a full width at half maximum (FWHM) Gaussian kernel of 5 mm, noise reduction, co‐registration of functional images to the structural image, and normalization to Montreal Neurological Institute (MNI) space. Subsequently, task and rest cycles were convolved with the hemodynamic response function to construct the general linear model (GLM). For each participant, a fixed‐effects higher‐level GLM analysis was performed, applying a threshold of *z* ≥ 3.1 and a corrected cluster *p* threshold of *p* < 0.05, based on Gaussian random field theory. Lateralization of brain activity was evaluated for each participant by calculating the Laterality Index (LI), which was computed using the following equation:

$$\mathrm{Laterality}\mathrm{Index}\left(\mathrm{LI}\right)=\frac{L-R}{L+R}$$

where L and R represent the number of activated voxels in Broca's area in the left and right hemispheres, respectively.

### 2.5 Statistical Analysis

Statistical analyses were performed to compare LI values across the object naming, word generation, and MTL tasks. Pairwise comparisons between tasks (object naming vs. word generation, object naming vs. MTL, and word generation vs. MTL) were conducted using appropriate inferential tests based on data distribution (independent‐samples *t*‐test or Mann–Whitney *U* test). All tests were two‐tailed, and statistical significance was set at *p* < 0.05. Where multiple pairwise comparisons were performed, *p*‐values were adjusted using Bonferroni correction. General statistical analyses were performed using IBM SPSS Statistics version 27.

## 3 Results

### 3.1 Participant Characteristics

The demographic and lesion‐location characteristics of the 25 enrolled patients are presented in Table 1.

**TABLE 1:** Patients’ characteristics.

Number	Age	Mass lesion location
1	57	Left temporal lobe
2	29	Left frontotemporal
3	34	Left parietal lobe
4	20	Left frontal lobe
5	26	Left parietal lobe
6	36	Right frontoparietal
7	59	Left frontoparietal
8	21	Left temporal lobe
9	23	Left temporal lobe
10	41	Left frontotemporal
11	54	Left temporal lobe
12	33	Right temporal lobe
13	44	Left frontal lobe
14	34	Right frontoparietal
15	55	left temporal lobe
16	47	Left temporal lobe
17	35	Left frontal lobe
18	47	Left frontoparietal
19	47	Left frontal
20	41	Right frontoparietal
21	18	Left motor cortex
22	59	Right temporoparietal
23	49	Left temporal lobe
24	37	Left frontoparietal
25	31	Left frontal lobe

### 3.2 Language

The CVR for all items ranged from 0.9 to 1, with a mean of 0.93 (SD: 0.09). The CVI for all items ranged from 0.8 to 1, with a mean of 0.94 (SD: 0.07). Among the 50 tests, 41 (82%) were considered feasible for the fMRI language task, as they met the criterion of more than two‐thirds of participants (less than 11 failed subjects) successfully producing an appropriate sentence in under 4 s (Table 2).

**TABLE 2:** The CVR, CVI, and number of failed subjects for each item of the Persian language paradigm for intraoperative stimulation.

Test number	1	2	3	4	5	6	7	8	9	10
**Sentence**	The fire burns	The horse runs	The ambulance carries	The pliers hold	The mirror shows	It is raining	The tiger looks	The brush scratches	The leave falls	The shovel digs
**CVR**	1	1	0.8	0.8	1	1	1	0.8	1	1
**CVI**	1	1	0.9	0.9	0.9	1	1	0.9	1	1
**Failed subjects**	2	0	13	13	5	8	3	17	1	11
**Test number**	**11**	**12**	**13**	**14**	**15**	**16**	**17**	**18**	**19**	**20**
**Sentence**	The police arrests	The fan spins	The pinguin stands	The gun shoots	The phone rings	The vacuum cleaner sweeps	The knife cuts	The eyes see	The hammer hits	The towel dries
**CVR**	0.8	1	1	0.8	1	0.8	1	1	1	0.8
**CVI**	0.8	0.9	1	0.8	1	0.8	0.9	1	0.9	0.9
**Failed subjects**	6	2	7	13	4	13	5	6	8	10
**Test number**	**21**	**22**	**23**	**24**	**25**	**26**	**27**	**28**	**29**	**30**
**Sentence**	The lobster bites	The sun is shining	The doctor examines	The button closes	The camera takes pictures	The tongue tastes	The dog barks	The star shines	The camel walks	The fish swims
**CVR**	0.8	1	1	0.8	1	0.8	1	1	1	1
**CVI**	1	0.9	1	0.8	1	0.8	1	0.9	1	0.9
**Failed subjects**	9	3	3	17	3	9	0	1	0	1
**Test number**	**31**	**32**	**33**	**34**	**35**	**36**	**37**	**38**	**39**	**40**
**Sentence**	The soap washes	The parrot talks	The eagle flies	The train lefts	The frog jumps	The scissor cuts	The kangaroo jumps	The pigeon flies	The kettle boils	The key opens
**CVR**	0.8	1	1	1	0.8	1	1	1	0.8	1
**CVI**	0.9	1	1	1	0.8	1	1	0.9	0.8	1
**Failed subjects**	13	3	2	5	5	2	3	2	7	3
**Test number**	**41**	**42**	**43**	**44**	**45**	**46**	**47**	**48**	**49**	**50**
**Sentence**	The cat fears	The sparrow sings	The sheep eats	The ears hear	The snake bites	The meter measures	The pencil writes	The nail grows	The lion seats	The refrigerator cools
**CVR**	1	1	0.8	0.8	1	0.8	0.8	1	1	1
**CVI**	1	0.9	1	0.9	1	1	0.9	1	1	1
**Failed subjects**	5	3	6	8	2	12	9	4	2	1

The present study evaluated item validity, feasibility (completion within 4 s), and preoperative fMRI activation patterns; intraoperative seizure incidence during direct cortical stimulation was not collected as a study outcome.

### 3.3 Imaging Findings

The results indicate significant activations across various regions, with the most pronounced response during object naming, particularly in the left precentral gyrus, inferior frontal gyrus (IFG), pars opercularis (PO), Broca, paracingulate gyrus, superior frontal gyrus (SFG), and premotor cortex (*z* = 7.53). For word generation, significant activations were identified bilaterally in the fusiform gyrus and Broca with a peak *z*‐score of 5.54. The MTL task exhibited its highest activation (*z* = 6.86) in both fusiform gyrus and inferior temporal gyrus (ITG). Table 3 presents a detailed analysis of brain activations observed during object naming, word generation, and MTL tasks.

**TABLE 3:** Brain activations in object naming, word generation and MTL tasks.

MNI coordinate			Cluster size	Region	Hemisphere	*z*‐score	Log10(*p*)
*x*	*y*	*z*					
**Object naming**							
40	−82	−20	13,164	Fusiform gyrus	R	5.98	66
−60	10	10	6719	Precentral gyrus, IFG, PO, Broca, paracingulate gyrus, SFG, premotor cortex	L	7.53	41.7
−32	−94	−12	6294	Cerebellum	L	5.49	39.8
46	20	−10	2825	Insula, Broca, IFG, PT, MFG, PO, precentral gyrus	R	5.35	22.8
**Word generation**							
28	50	14	14,771	Pallidum, putamen, precentral gyrus, operculum, postcentral gyrus, Broca	L & R	4.62	38.6
50	−64	−26	9572	Fusiform gyrus, Broca	L & R	5.54	28.4
−48	−32	42	469	SG, postcentral gyrus, IPL	L	4.27	2.01
−24	−58	40	383	Angular gyrus, SG	L	3.92	1.5
**MTL**							
−34	−86	−20	23,432	Fusiform gyrus, ITG	L & R	6.86	94.4
0	12	52	20,272	Paracingulate gyrus, premotor cortex, superior frontal gyrus, SMA, operculum, IFG, PT, PO, Broca, precentral gyrus, MFG	L & R	6.15	85.6

IFG: inferior frontal gyrus, PO: pars opercularis, SFG: superior frontal gyrus, PT: pars triangularis, MFG: middle frontal gyrus, SG: supramarginal gyrus, IPL: inferior parietal lobule, ITG: inferior temporal gyrus, SMA: supplementary motor area.

**FIGURE 2:**
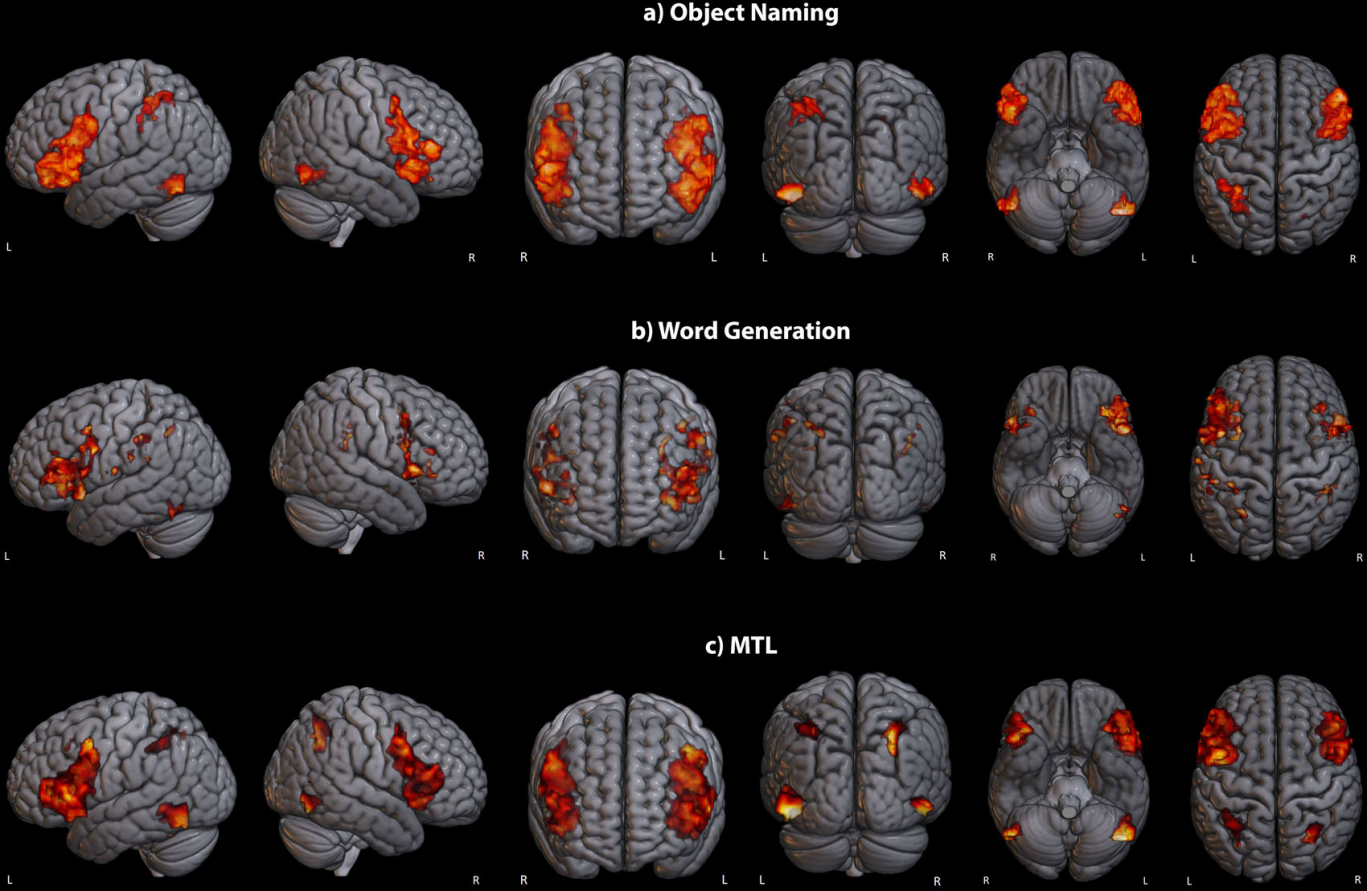
Activation maps of language‐related regions during (a) object naming, (b) word generation, and (c) MTL tasks.

Analyses of language‐related region activations were performed to reveal the importance of these areas in facilitating various aspects of language processing. Table 4 and Figure 2 show the activation of language‐related regions during object naming, word generation, and MTL tasks.

**TABLE 4:** Activation characteristics of language‐related regions during object naming, word generation, and MTL tasks.

Region	Object naming				Word generation				Multi‐task language			
	Right		Left		Right		Left		Right		Left	
	*Z* (mean ± SD)	*N* Voxels	*Z* (mean ± SD)	*N* Voxels	*Z* (mean ± SD)	*N* Voxels	*Z* (mean ± SD)	*N* Voxels	*Z* (mean ± SD)	*N* Voxels	*Z* (mean ± SD)	*N* Voxels
Angular	NaN	0	3.51 ± 0.25	3	NaN	0	2.66 ± 0.22	2	3.50 ± 0.24	110	3.53 ± 0.26	102
IFG	3.56 ± 0.31	1285	3.67 ± 0.36	1672	2.73 ± 0.30	633	2.75±0.29	1411	3.91 ± 0.47	1816	4.18 ± 0.57	2524
Ventral Premotor	3.58 ± 0.32	1314	3.63 ± 0.35	1771	2.72 ± 0.30	648	2.72 ± 0.28	1615	3.93 ± 0.53	1772	4.12 ± 0.58	2929
Supramarginal gyrus	NaN	0	3.33 ± 0.17	163	2.62 ± 0.25	57	2.62 ± 0.30	403	3.27 ± 0.13	9	3.49 ± 0.25	513
STG	NaN	0	NaN	0	NaN	0	NaN	0	NaN	0	3.13 ± 0.00	1
MTG	3.42 ± 0.23	50	3.29 ± 0.21	13	NaN	0	2.61 ± 0.33	9	3.35 ± 0.19	24	3.73 ± 0.46	340

**FIGURE 3:**
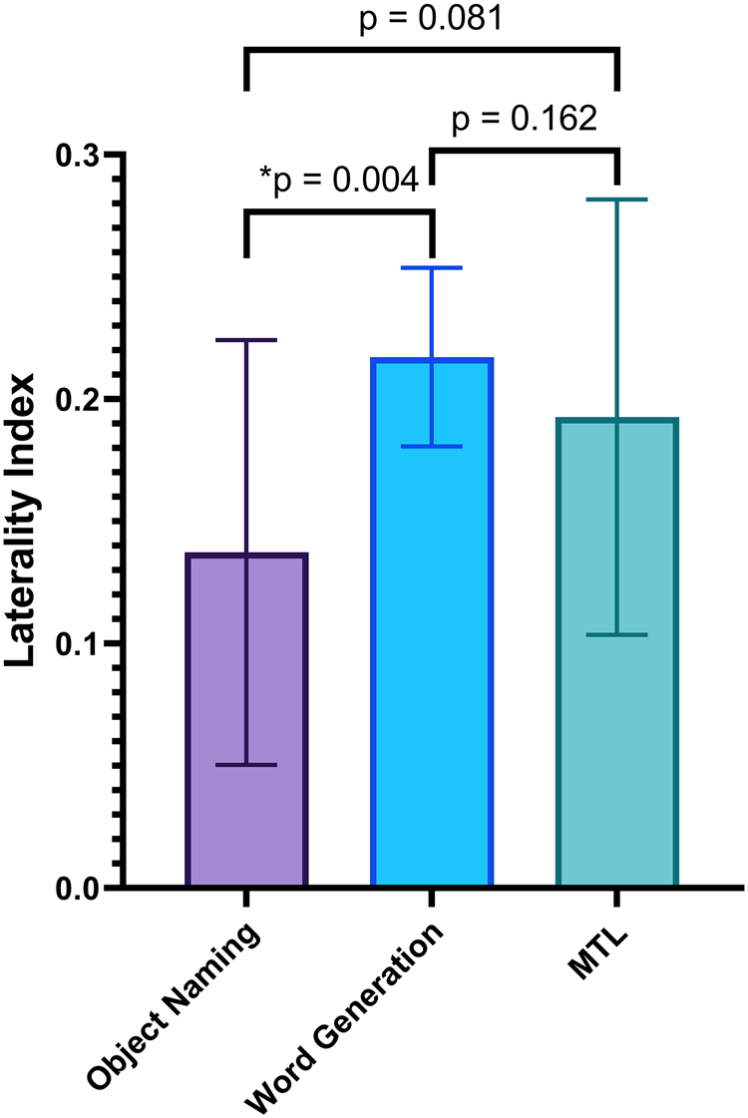
Laterality Index (LI) across object naming, word generation, and multi‐task language (MTL) tasks. Pairwise comparisons are shown above the plots (object naming vs. word generation: *p* = 0.004; object naming vs. MTL: *p* = 0.081; word generation vs. MTL: *p* = 0.162). Asterisk indicates statistical significance (*p < 0.05).

Table 5 and Figure 3 display the Laterality Index for object naming, word generation, and MTL tasks, along with *p*‐values for pairwise comparisons. The results indicate a significant difference between object naming and word generation, while the comparisons involving MTL do not show statistical significance.

**TABLE 5:** Laterality Index for comparative analysis across various tasks.

Laterality Index (mean ± SD)			*p*‐value		
Object naming	Word generation	MTL	Object naming vs. word generation	Object naming vs. MTL	Word generation vs. MTL
0.13 ± 0.08	0.21 ± 0.03	0.19 ± 0.08	0.004	0.081	0.162

## 4 Discussion

The study aimed to create a Persian intraoperative language paradigm that assesses key language functions by combining object naming and word generation tasks. Content validity showed that all items were valid for Persian use. Each item was designed to elicit a grammatically correct sentence in under 4 s to minimize seizure risks from intraoperative DCS. Ultimately, 41 of the 50 items were deemed feasible, forming the final version of the paradigm. In fMRI analyses, the MTL task activated more language‐related regions compared to the object naming and word generation tasks. Additionally, the LI for the MTL task did not show a significant difference when compared to either of the other two tasks.

Four established assessments have been specifically designed for the intraoperative evaluation of language during awake craniotomies. The Dutch Linguistic Intraoperative Protocol (DuLIP) (De Witte et al. 2015a,b) comprises 17 tasks that evaluate various language domains, including phonology, semantics, syntax, and morphology. These tasks assess multiple functions such as speech production, naming, comprehension, reading, and repetition, as well as different speech systems, including articulation and motor programming. The normative data for these tasks are derived from Dutch linguistic databases, such as CELEX, SUBTLEX‐NL, and the Positie woordenboek.

Rofes et al. (2015a, b) established standardized tests for object and verb naming specifically for intraoperative language assessment in the Italian language. In addition to these two tasks, an automated formulaic speech task (counting from 0 to 10) was utilized to facilitate a comprehensive evaluation of language within a relatively brief timeframe. In contrast to the DuLIP and the aforementioned tests, the current study does not provide normative data for the developed language paradigm, underscoring the necessity to address this gap in future research.

Another brain mapping assessment conducted during awake craniotomy was developed by Dragoy et al. (2016) in the Russian language. The Russian Intraoperative Naming Test similarly comprises tasks for object naming and verb naming. Additionally, a normative report based on healthy participants is available for this test. However, the authors did not provide details regarding the procedures for establishing the test's validity and reliability. Likewise, the current study is also devoid of reliability data.

All three preceding assessments incorporate two or more evaluation tasks. A novel single‐language paradigm, which combines picture naming and sentence generation tasks, was developed by Rosengarth et al. (2021) in German. This investigation demonstrated that the innovative language paradigm effectively monitors critical language functions intraoperatively, thereby potentially reducing adverse postoperative neuropsychological outcomes. The current study employed a similar paradigm in Persian, further supporting the notion that this novel language framework is safe and provides a more comprehensive assessment of language abilities.

The implementation of sentence generation tasks presents temporal limitations, potentially compromising their feasibility in time‐constrained intraoperative protocols (Rofes and Miceli 2014; Rosengarth et al. 2021). The temporal efficiency of our protocol represents a significant methodological advantage compared to conventional sentence completion paradigms, which frequently exceed temporal constraints and present implementation challenges in time‐sensitive surgical contexts.

The observed patterns of brain activation during object naming, word generation, and MTL processing likely stem from a combination of factors, including task demands, cognitive control mechanisms, and the involvement of a default language network. Compared to traditional naming and word generation tasks, the MTL task demonstrated more activation across language‐related cortical regions. This may be attributed to rising task demands and reduced response time. Higher cognitive demands typically lead to greater brain activation, while limited time for evaluating picture–word matches necessitates quicker responses, resulting in enhanced neural engagement (Fridriksson and Morrow 2005; Zhang et al. 2018).

The MTL task exhibited activation in several critical regions associated with language processing, particularly the left IFG, premotor cortex, and inferior parietal lobule (IPL). These regions are frequently linked to tasks involving both action naming and word generation. Notably, the left IFG is associated with lexical retrieval and language production, whereas the premotor cortex and IPL play significant roles in action comprehension and execution (Wang et al. 2022a,b).

The convergence of activated regions observed in the MTL, object naming, and word generation tasks can be ascribed to their common dependence on semantic processing and motor planning. Nevertheless, variations may occur due to the distinct requirements of each task. For example, object naming predominantly involves visual recognition and lexical access, whereas word generation necessitates a more intricate synthesis of semantic knowledge and motor planning (Wang et al. 2022a,b). This indicates that, although these tasks engage overlapping neural substrates, the specific context and demands of each task influence the extent of activation within these regions.

Prior researches investigating paradigms that integrate object naming and word generation tasks have yielded similar results. Specifically, studies have consistently demonstrated that both tasks activate overlapping regions in the left IFG, premotor cortex, and IPL, indicating a shared neural foundation for language processing associated with actions and objects (Ojemann et al. 2002; Kuan et al. 2024). Additionally, studies employing multi‐voxel pattern analysis (MVPA) have identified distinct activation patterns within these common regions for action and word generation tasks. This finding suggests that, although these tasks utilize shared neural resources, they engage these resources in different ways based on the specific demands of each task (Wang et al. 2022a,b).

The comparable LI between the MTL paradigm and conventional tasks suggests preserved hemispheric specialization patterns, supporting the methodological validity of this integrated protocol. The observed lateralization equivalence corroborates extant literature demonstrating the efficacy of composite assessment paradigms in delineating neural substrates of language processing (Kuan et al. 2024).

Our findings add to the expanding literature that underscores the significance of integrating various linguistic tasks to clarify the underlying neural mechanisms. The convergence between object naming and word generation tasks has been observed in multiple contexts; for instance, cortical stimulation mapping studies have demonstrated that both tasks frequently activate similar cortical regions, although different areas may also be recruited based on the specific linguistic requirements (Ferré et al. 2020; Ojemann et al. 2002).

Furthermore, our results align with studies that emphasize the role of the left IFG in both action‐related language processing and broader language production. The activation of this region during both object naming and word generation indicates a strong network for semantic retrieval that is responsive to the contextual demands of the tasks (Wang et al. 2022a,b).

The 4‐s completion constraint in our paradigm was selected in accordance with recommendations for direct cortical stimulation mapping to reduce the likelihood of stimulation‐related adverse events, including seizures (Kayama 2012). However, intraoperative seizure prevalence was not prospectively recorded in the present study; therefore, our findings do not directly demonstrate seizure reduction and should be interpreted as supporting feasibility within a time‐constrained mapping framework rather than clinical seizure prevention efficacy.

This study has several limitations. First, the sample size was relatively small, which may limit the generalizability of the findings. Second, the lesion locations were heterogeneous (including frontal, temporal, parietal, and frontoparietal regions), and this heterogeneity may have influenced activation patterns, task performance, and laterality estimates. Third, language function is organized as a distributed network involving cortical and subcortical pathways rather than isolated focal cortical regions; therefore, region‐specific activation differences should be interpreted within a network‐based framework. Finally, the lack of fMRI data from healthy participants prevented direct validation of the MTL protocol against a control group and limited our ability to establish normative activation and lateralization patterns.

## 5 Conclusions

This study successfully developed a Persian intraoperative language paradigm that effectively assesses critical language functions through a combination of object naming and word generation tasks. The findings demonstrate that the paradigm is both valid and feasible, with a significant proportion of tasks meeting the established criteria for intraoperative use. Notably, the results indicate robust activation across various language‐related regions during the tasks, particularly in the left inferior frontal gyrus and other key cortical areas associated with language processing. While the study highlights the potential of this novel approach to minimize postoperative neuropsychological risks, it also emphasizes the need for further research to establish normative data and enhance the reliability of the paradigm. Overall, these contributions underscore the importance of integrating diverse linguistic assessments in the context of awake craniotomies to improve outcomes for patients undergoing neurosurgical procedures.

## Author Contributions

**AT**: conceptualization, methodology, investigation, visualization, validation, and writing – review and editing. **MJ**: conceptualization, methodology, software, data curation, writing – original draft, investigation, visualization, validation, formal analysis, and writing – review and editing. **VV**: conceptualization, methodology, writing – original draft, and investigation. **ES**: conceptualization, methodology, writing – original draft, and investigation. **MM**: software, writing – original draft, investigation, visualization, validation, formal analysis, and writing – review and editing. **ZO**: software, data curation, investigation, visualization, validation, and formal analysis. **PD**: conceptualization and methodology. **MA**: conceptualization and methodology. **MO**: conceptualization, methodology, investigation, visualization, validation, writing – review and editing, supervision, and project administration.

## Funding

The authors have nothing to report.

## Ethics Statement

Written consent was obtained from all the participants with potentially identifiable images or data. The study was approved by the Ethics Committee of the Iran University of Medical Sciences.

## Conflicts of Interest

The authors declare that they have no conflicts of interest.

## Supporting information




**Supplementary Table**: brb371324‐sup‐0001‐Table.docx

## Data Availability

This article contains all the data produced or analyzed during this investigation. Further inquiries should be forwarded to the corresponding author.
