# Supplementary material for: Persian Paradigm for Language Cortical Mapping: Development, Feasibility, and Evaluation
Source: Brain Behav. 2026 Mar 31;16(4):e71324. doi: 10.1002/brb3.71324 (PMC13112009; doi:10.1002/brb3.71324)
Supplement: Supplementary file 1 — Supplementary Table: brb371324‐sup‐0001‐Table.docx [file BRB3-16-e71324-s001.docx]

| **Supplementary Table.** Finalized Persian stimuli used in the multi-task language (MTL) paradigm (original Persian language list) | | |
| --- | --- | --- |
| Persian Stimulus | | English Translation |
| Image Concept + Verb | Sentence Frame Used |  |
| 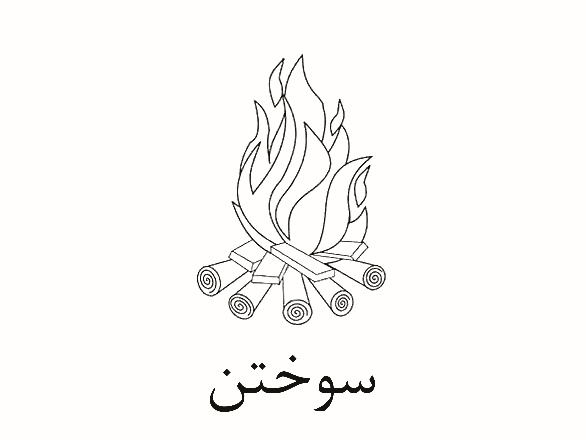 | آتش می‌سوزد | The fire burns |
| 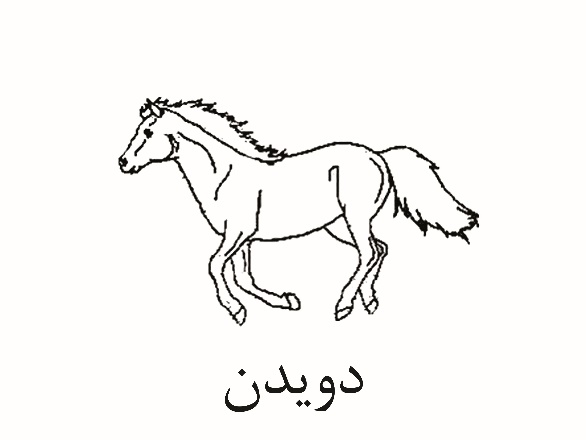 | اسب می‌دود | The horse runs |
| 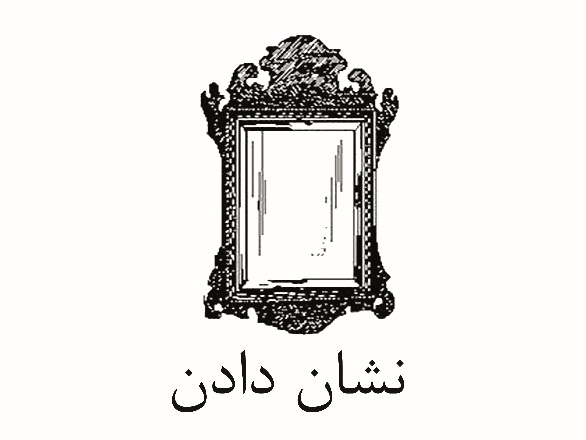 | آینه نشان می‌دهد | The mirror shows |
| 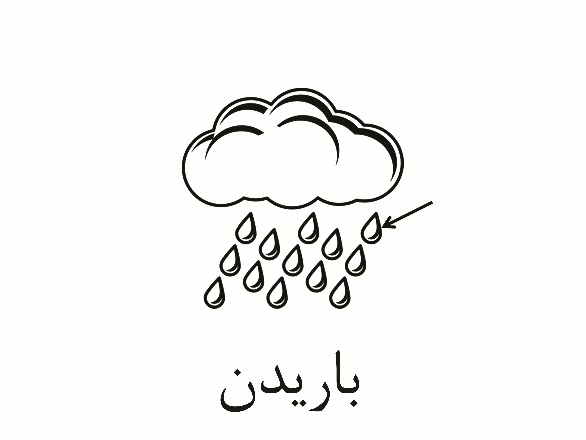 | باران می‌بارد | It is raining |
| 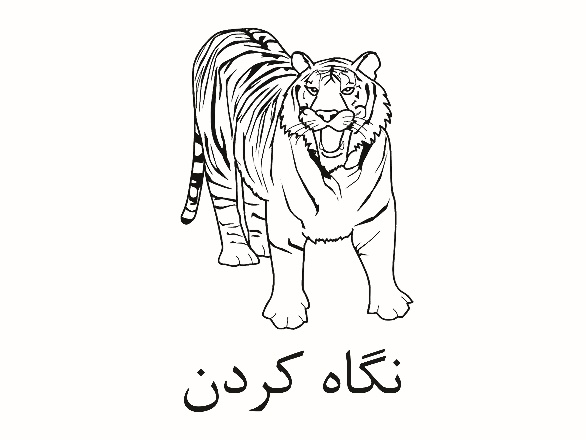 | ببر نگاه می‌کند | The tiger looks |
| 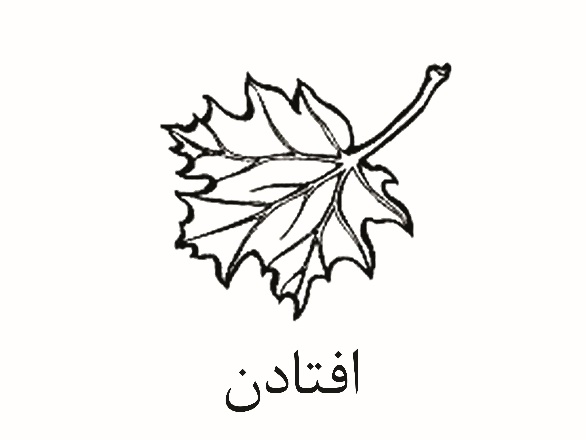 | برگ‌ می‌ریزد | The leave falls |
| 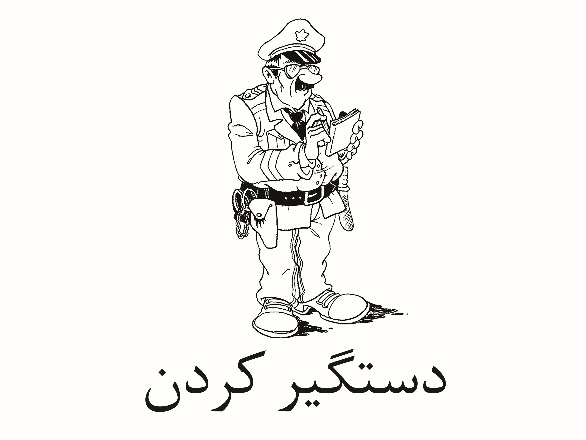 | پلیس دستگیر می‌کند | The police arrests |
| 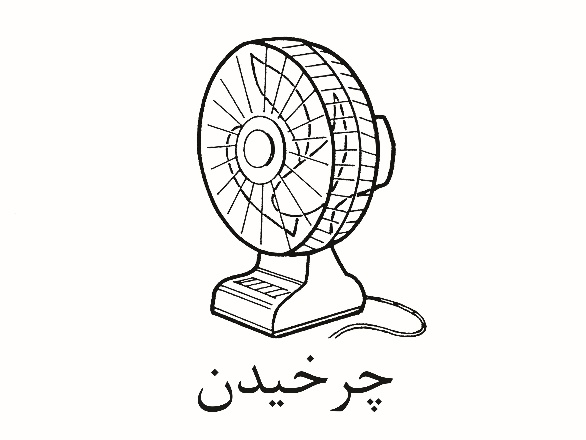 | پنکه می‌چرخد | The fan spins |
| 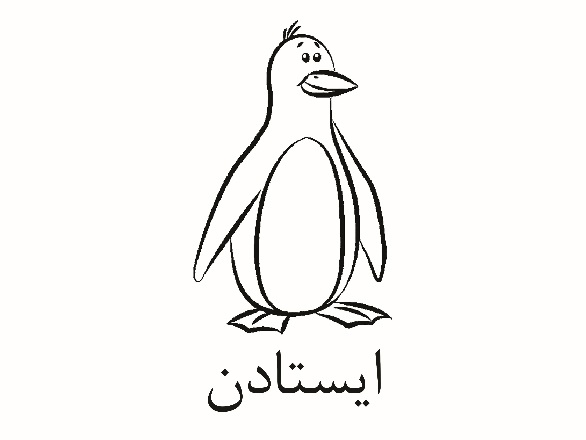 | پنگوئن می‌ایستد | The pinguin stands |
| 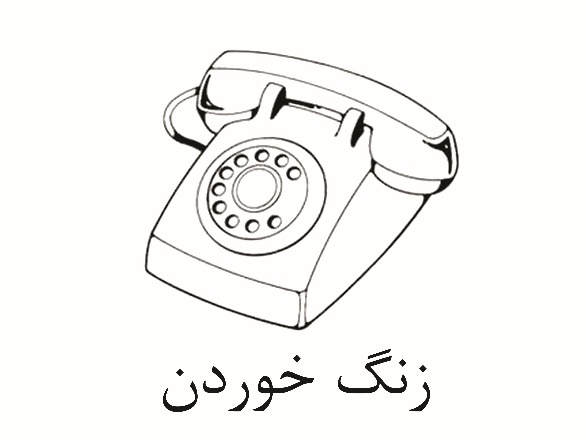 | تلفن زنگ می‌خورد | The phone rings |
| 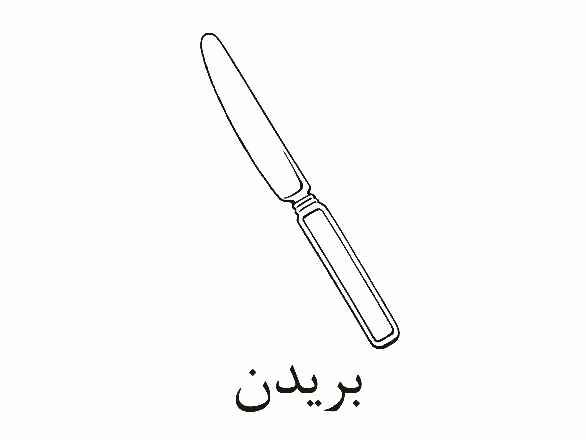 | چاقو می‌برد | The knife cuts |
| 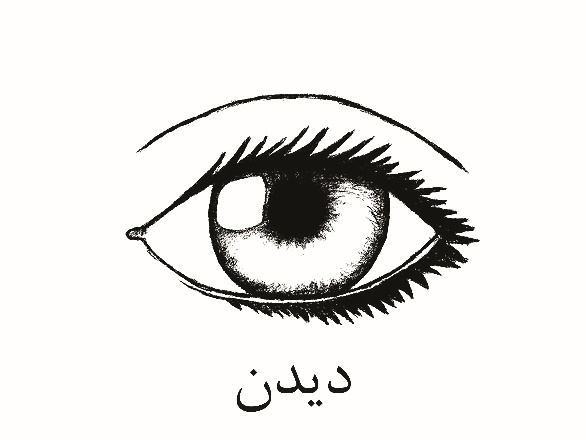 | چشم‌ها می‌بینند | The eyes see |
| 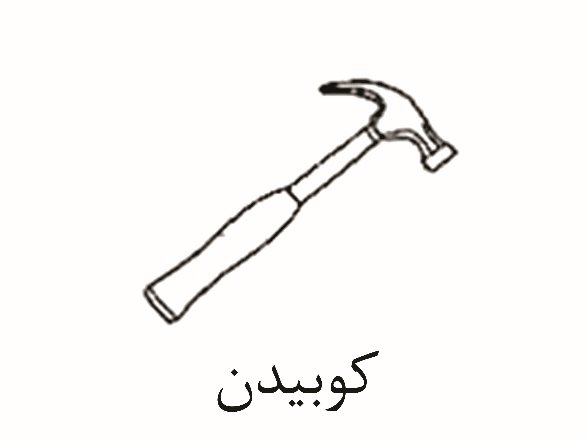 | چکش می‌کوبد | The hammer hits |
| 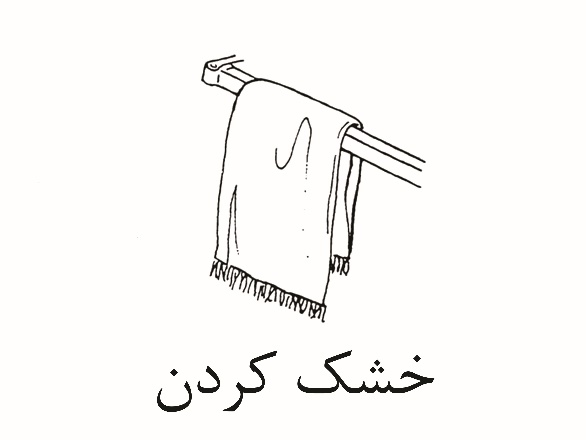 | حوله خشک می‌کند | The towel dries |
| 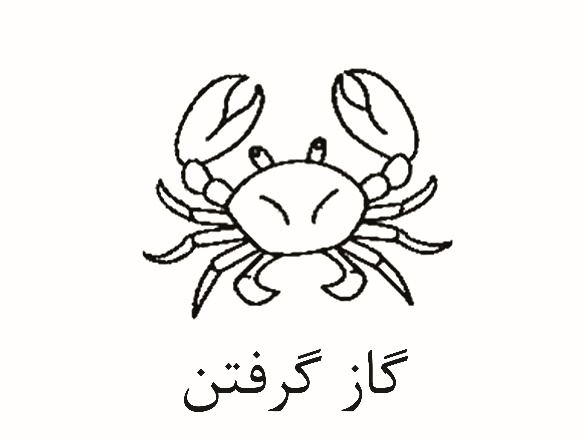 | خرچنگ گاز می‌گیرد | The lobster bites |
| 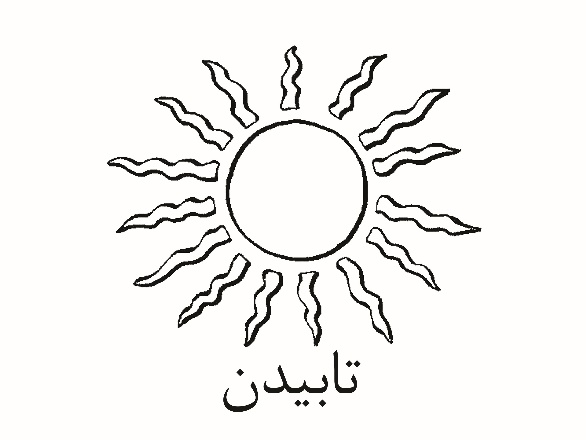 | خورشید می‌تابد | The sun is shining |
| 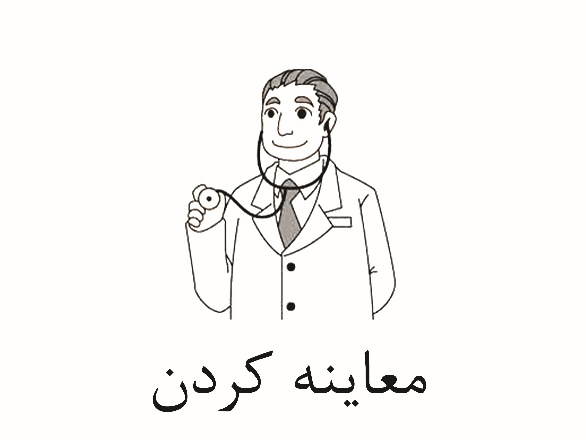 | دکتر معاینه می‌کند | The doctor examines |
| 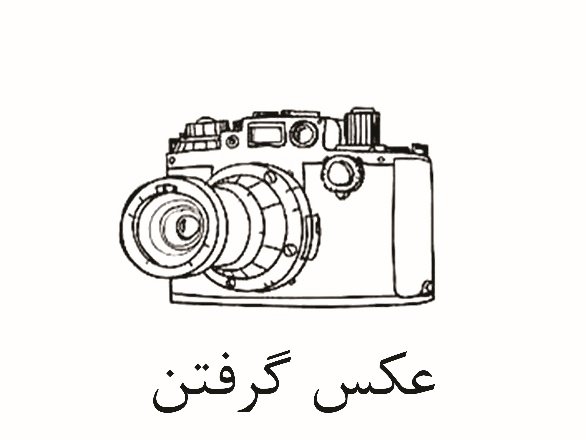 | دوربین عکس می‌گیرد | The camera takes pictures |
| 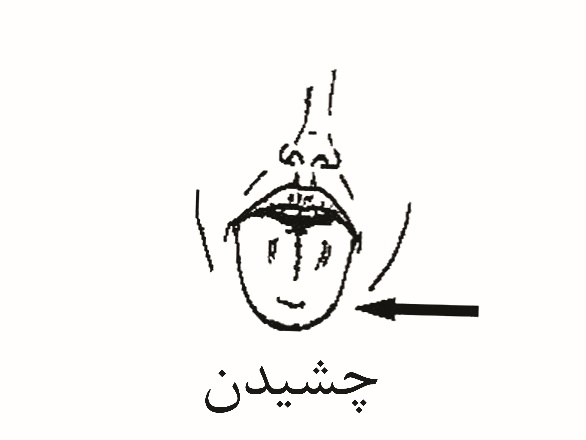 | زبان می‌چشد | The tongue tastes |
| 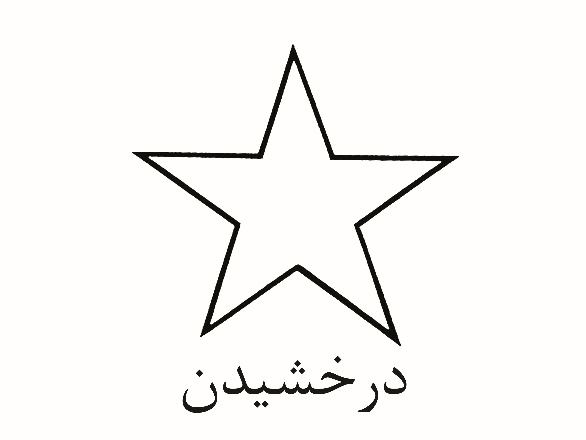 | ستاره می‌درخشد | The star shines |
| 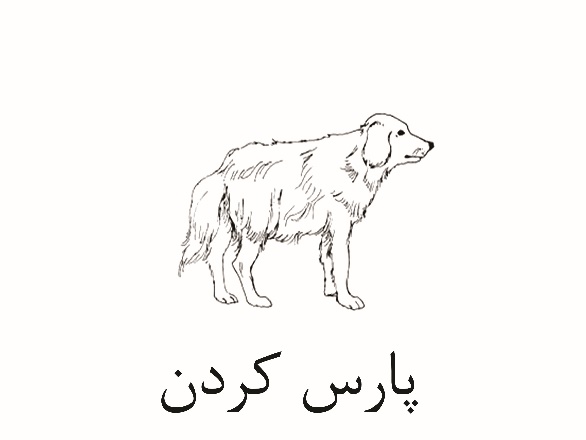 | سگ پارس می‌کند | The dog barks |
| 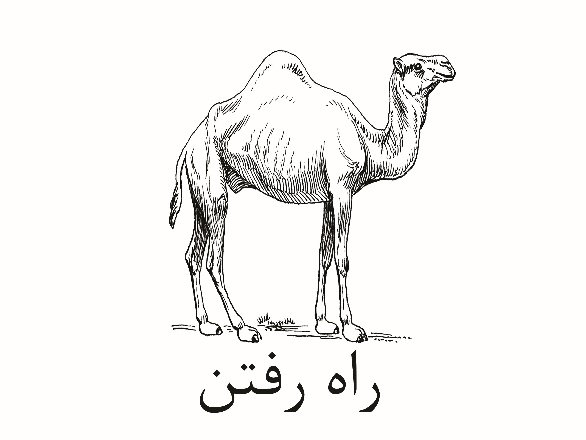 | شتر راه می‌رود | The camel walks |
| 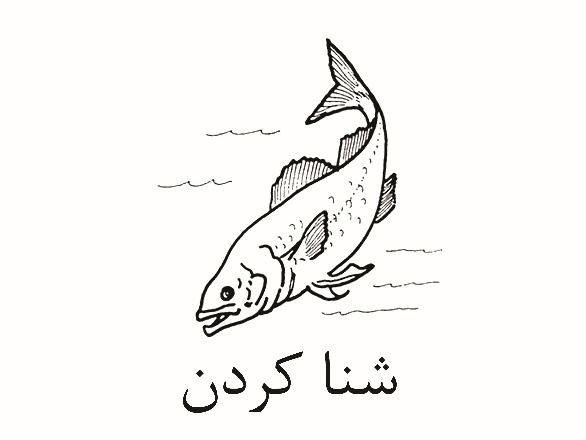 | ماهی شنا می‌کند | The fish swims |
| 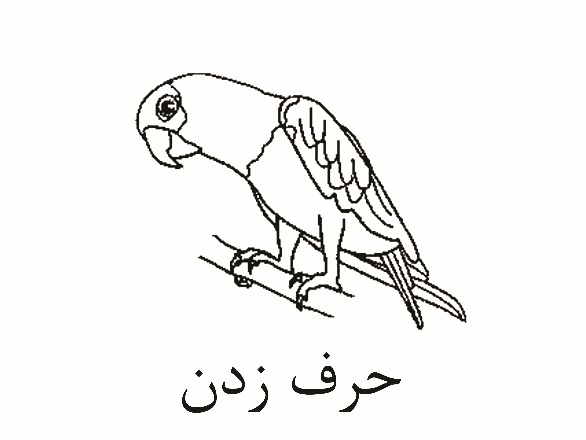 | طوطی حرف می‌زند | The parrot talks |
| 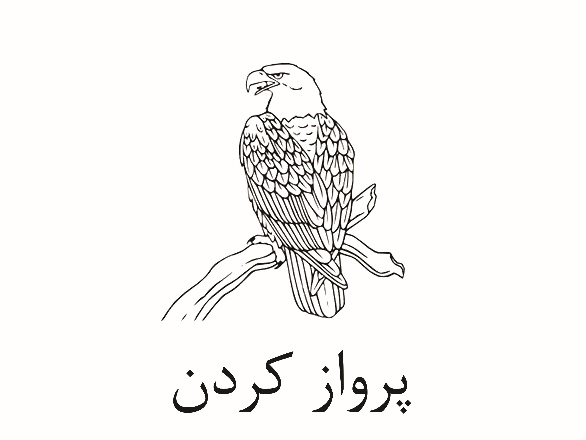 | عقاب پرواز می‌کند | The eagle flies |
| 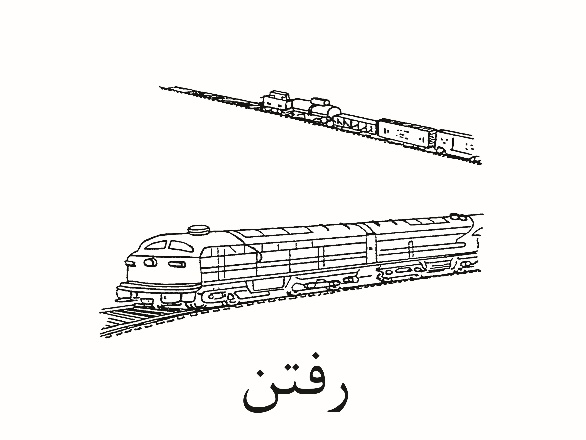 | قطار می‌رود | The train lefts |
| 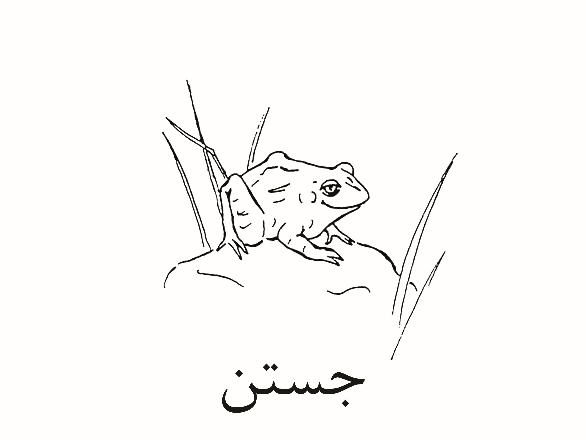 | قورباغه می‌جهد (می‌پرد) | The frog jumps |
| 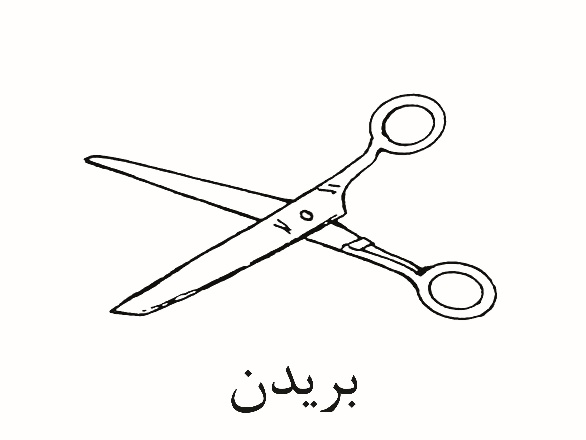 | قیچی می‌برد | The scissor cuts |
| 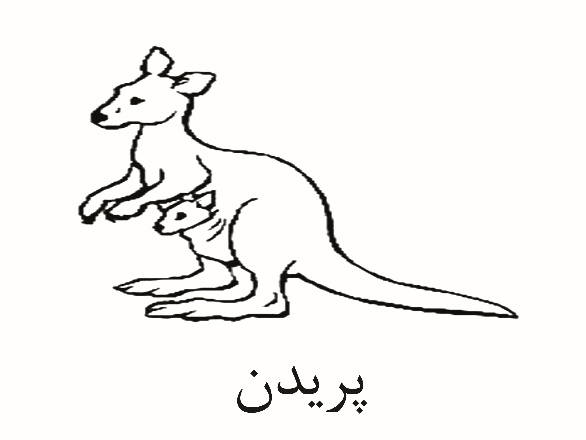 | کانگورو می‌پرد | The kangaroo jumps |
| 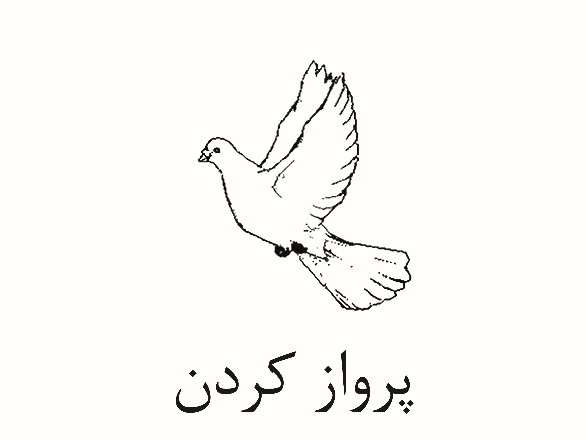 | کبوتر پرواز می‌کند | The pigeon flies |
| 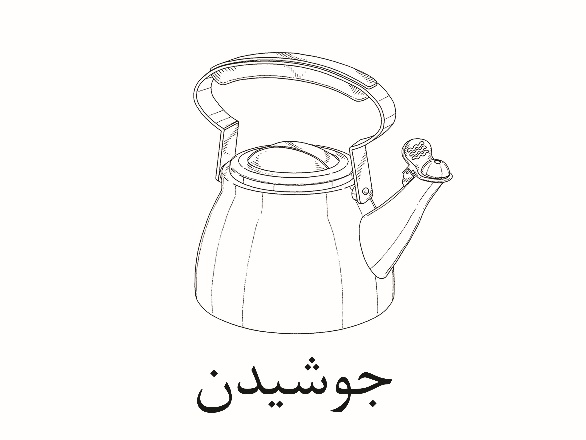 | کتری می‌جوشد | The kettle boils |
| 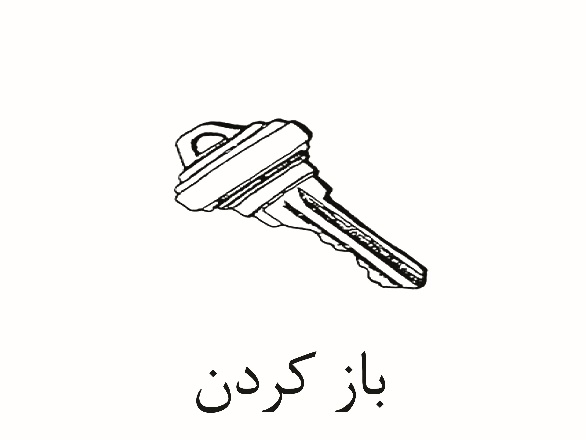 | کلید باز می‌کند | The key opens |
| 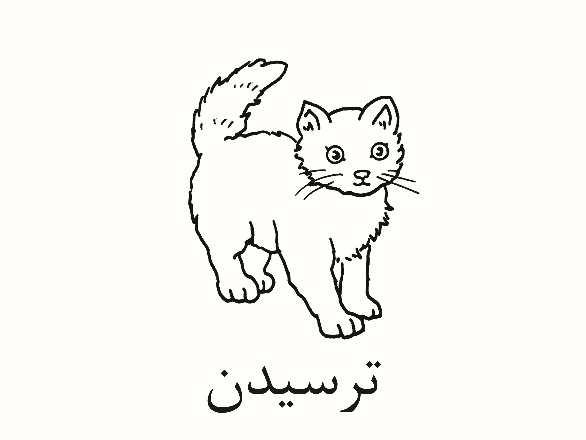 | گربه می‌ترسد | The cat fears |
| 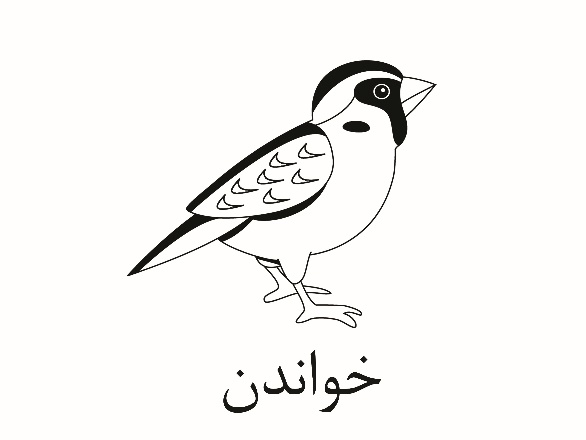 | گنجشک آواز می‌خواند | The sparrow sings |
| 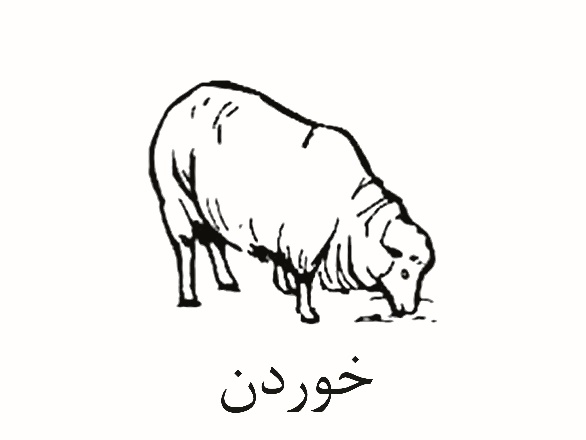 | گوسفند می‌خورد | The sheep eats |
| 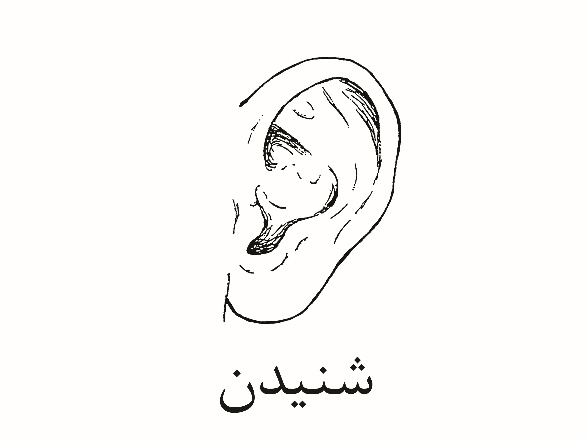 | گوش‌ها می‌شنوند | The ears hear |
| 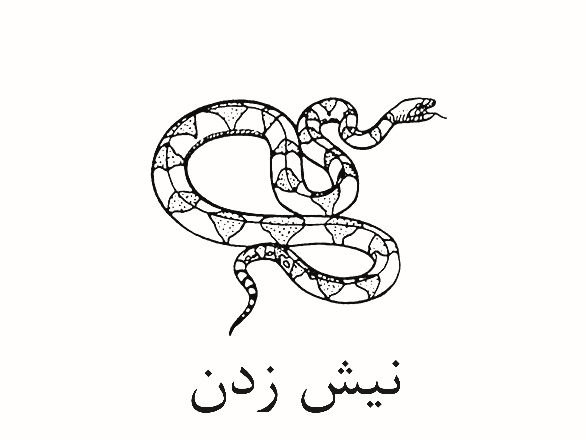 | مار نیش می‌زند‌ | The snake bites |
| 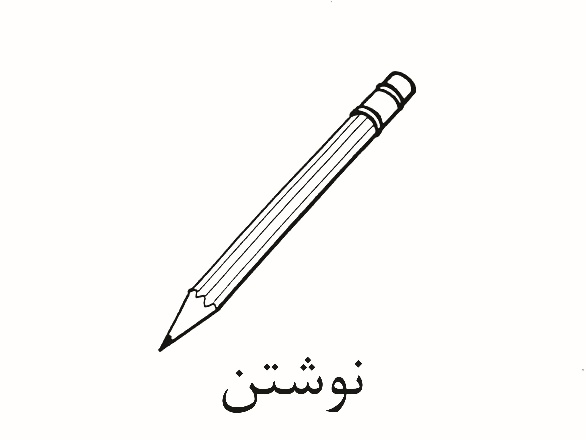 | مداد می‌نویسد | The pencil writes |
| 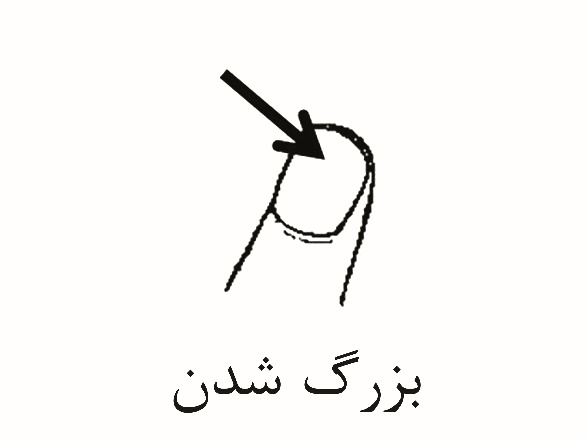 | ناخن بزرگ می‌شود | The nail grows |
| 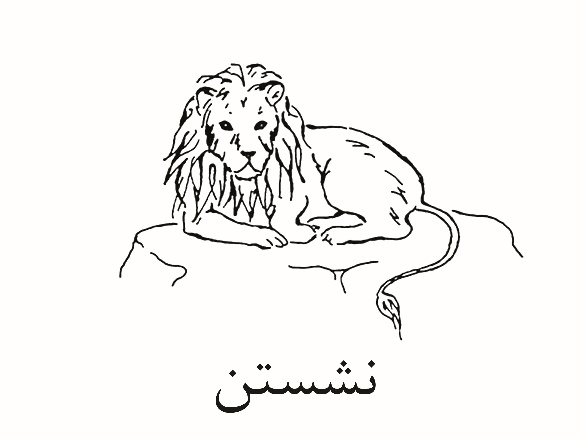 | شیر می‌نشیند | The lion seats |
| 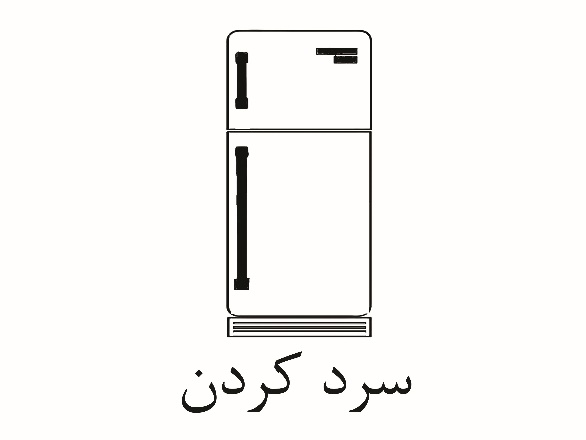 | یخچال سرد می‌کند | The refrigerator cools |
